# Neuroendocrine Neoplasm of the Breast Presenting as a Liver Metastasis: A Rare Diagnostic Challenge

**DOI:** 10.7759/cureus.16860

**Published:** 2021-08-03

**Authors:** Muhammad Masab, Alexander Gross, Melina Flanagan, Richard Goldberg, Midhun Malla

**Affiliations:** 1 Hematology and Medical Oncology, West Virginia University, Morgantown, USA; 2 Pathology and Laboratory Medicine, West Virginia University, Morgantown, USA

**Keywords:** neuroendocrine neoplasm, breast cancer pathology, neuroendocrine breast tumor, captem, metastatic liver lesion

## Abstract

We present a case of a 58-year-old female who presented initially to an outside institution with abdominal pain and was diagnosed on liver biopsy with a well-differentiated neuroendocrine tumor of an unknown primary source. She was referred to our academic institution for a second opinion after disease progression on the initial chemotherapy regimen. Through additional evaluation, diagnostics, and multi-disciplinary tumor board discussion she was diagnosed with metastases from a well-differentiated neuroendocrine neoplasm of the breast (NENB). Consequently, her treatment plan was modified leading to significant clinical and radiological improvement.

## Introduction

Well-differentiated neuroendocrine neoplasms most commonly originate from the small bowel, pancreas, and lung. Metastases of NENB to the viscera are uncommon but well documented [1]. A neuroendocrine neoplasm of the breast (NENB) is rare but may benefit from treatment according to guidelines applied to invasive breast carcinoma [2]. Diagnosis of a NENB is challenging due to subtle morphology and lack of routine immunostaining [2]. Confirmation of histogenesis of primary and metastatic sites in NENB is vital for appropriate management. As illustrated by this case, cross-disciplinary communication is imperative for proper work-up, diagnosis, and treatment in these patients.

## Case presentation

A 58-year-old white female presented to our cancer center for worsening metastatic disease due to a well-differentiated neuroendocrine tumor (NET) in the liver, despite four cycles of chemotherapy. Prior to her referral, she had initially presented to an outside hospital with abdominal pain, nausea, and vomiting. She denied symptoms of diarrhea or flushing. A CT scan with contrast done at outside facility revealed a 1.7-cm left outer quadrant breast mass, 2.5 cm left axillary lymph node, several peripherally enhancing liver lesions at largest measuring 6.2 cm. Pathology from a CT-guided biopsy of liver lesion done at the outside institute was reported as a high-grade well-differentiated NET. Notably, the breast mass was not sampled, and no somatostatin receptor-based imaging was pursued to determine the primary site of origin prior to initiating chemotherapy. She was referred to our academic tertiary care center after the progression of disease in the liver, abdominal lymph nodes, and thoracic spine. Examination done by oncologists at our institute revealed an ill-defined mass in the left lateral breast and a 2.0 cm lymph node in the left axilla.

**Figure 1:**
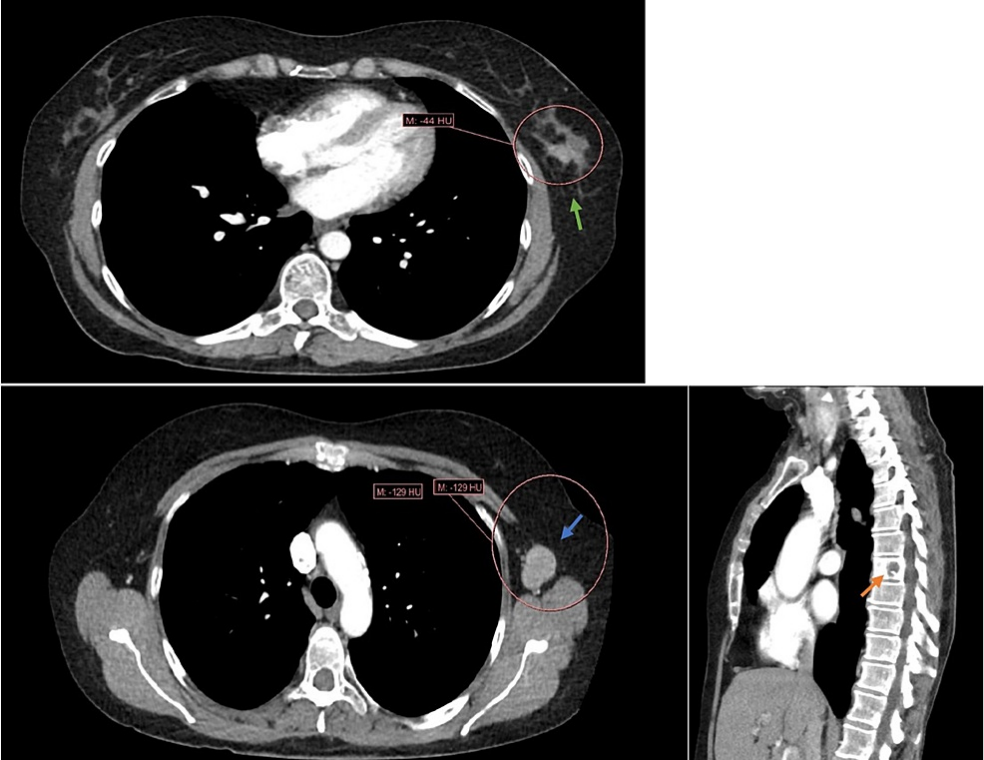
CT scan chest/abdomen/pelvis showing a mass in left breast measuring 2.6 x 2 cm (green arrow), left axillary lymphadenopathy (blue arrow), and a lytic lesion in T7 vertebral body concerning osseous metastasis (orange arrow).

CT scan performed at our institution demonstrated a 2.6 cm left breast mass, multiple enhancing masses in the liver, the largest measuring 6.7 cm, and a T7 lytic lesion with pathologic fracture (Figure 1).

**Figure 2:**
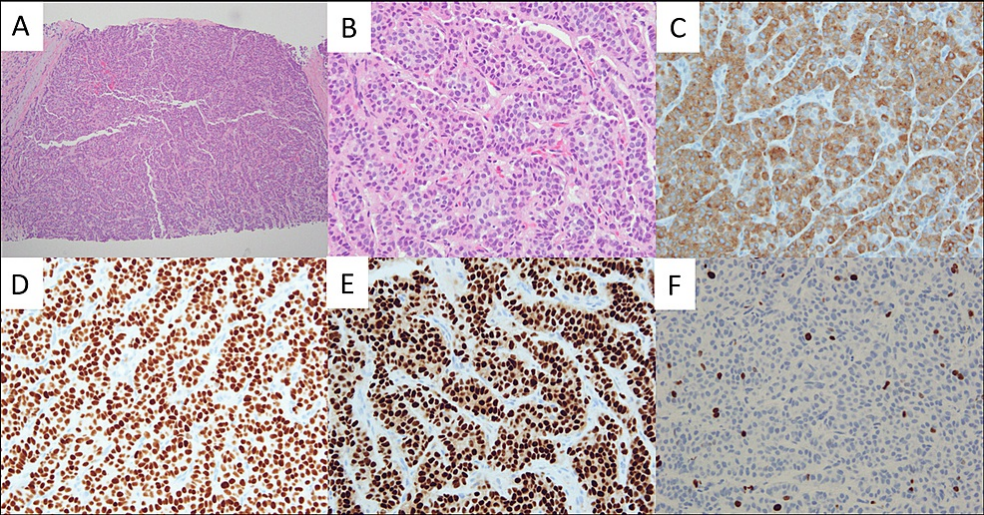
(A) Sheets of bland tumor cells in a nested pattern, H&E, 100x. (B) Round to oval nuclei with granular powdery chromatin, H&E, 400x. (C) Tumor cells were immunoreactive for synaptophysin (as well as chromogranin, not pictured), 400x. (D) GATA3, 400x. (E) ER, 400x. (F) Ki-67, 14%, 400x. ER - estrogen receptor

Breast biopsy was reported as Nottingham grade 3 invasive ductal carcinoma, and axillary lymph node was positive for metastasis. Of note, these were assessed by the pathologist prior to the liver biopsy being accessioned in our system, and without knowledge of the patient’s additional tumor burden. After discussion at the multidisciplinary breast tumor board, the breast biopsy was re-reviewed, and the liver biopsy was obtained for additional staining and review. The breast biopsy showed sheets of uniform nested cells with round to oval nuclei with vaguely powdery chromatin. A panel of immunostains was then performed, which showed that the tumor cells were immunoreactive for GATA3, synaptophysin (greater than 90% of tumor cells), chromogranin (greater than 90% of tumor cells), and estrogen receptor (ER). They were negative for glypican-3 and TTF-1. Ki-67 proliferation index was 14% (Figures 2A-2F).

**Figure 3:**
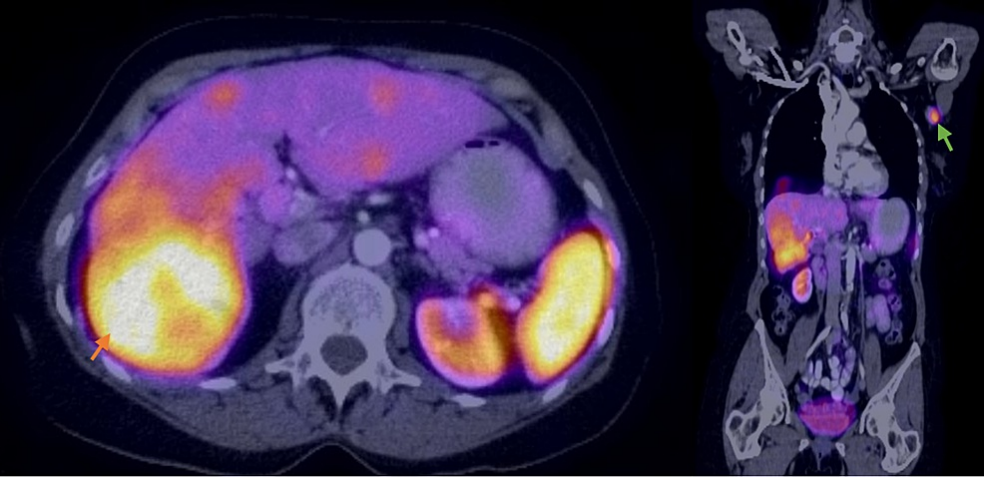
Gallium-68 dotatate PET-CT showing increased radiotracer uptake in liver (orange arrow) and left axillary lymph node (green arrow). PET-CT - Positron emission tomography–computed tomography

A review of the outside liver biopsy showed epithelioid tumor cells in nests and trabeculae with uniform nuclei. There were areas of necrosis and evident mitoses with Ki-67 of 30%. Tumor cells were immunoreactive for CK7, chromogranin, synaptophysin, and GATA3, and negative for HSA, glypican, and TTF-1. Gallium-68 dotatate positron emission tomography-computed tomography (PET-CT) performed at our site demonstrated increased uptake in left breast mass and ipsilateral axillary lymph node, liver lesions, hilar and mediastinal lymph nodes, left pleural-based chest wall nodule, and right thyroid. T7 lesion did not show increased uptake (Figure 3).

Serum chromogranin level was elevated at 243 ng/mL (normal < 93 ng/mL). Twenty-four hours urinary 5-hydroxy indoleacetic acid (5-HIAA) level was within normal limits. Given the spine metastasis, serum calcium level was checked and was within normal limits. Oncologists at the outside institution had previously started chemotherapy with carboplatin and etoposide. Etoposide was replaced with irinotecan due to a severe allergic reaction. Clinical disease progression was noted in the liver and thoracic spine after the completion of four cycles and was switched to oral everolimus. After a second opinion and definitive diagnosis around four months after the start of initial chemotherapy, our oncologists switched everolimus to lanreotide (somatostatin analog) and a chemotherapy combination of Capecitabine and Temozolomide (CAPTEM) [3]. She tolerated this regimen well except for grade 2 palmar-plantar erythrodysesthesia and neutropenia requiring one-time dose reduction. 

She continues to show a good response to the CAPTEM regimen and is enjoying an excellent quality of life. Her most recent MRI of the abdomen and Gallium-68 dotatate PET-CT showed a 63% reduction in the size of liver mass from 6.7 cm to 2.5 cm and decreased radiotracer uptake in both the primary breast mass and metastatic sites respectively. Interestingly, her circulating tumor DNA was assessed recently and was negative, which is suggestive of the absence of circulating tumor burden. She is currently on a chemotherapy break and continues to be on lanreotide injections. We continue to monitor her metastatic disease by checking circulating tumor DNA and Gallium-68 dotatate PET-CT scan every three months with a plan to re-treat her with a CAPTEM regimen or to consider her for liver-directed therapy upon disease progression. 

## Discussion

A broad differential for a patient with masses in multiple organ sites includes synchronous primaries and metastases. In this patient, the diagnosis of invasive ductal carcinoma of the breast was made at the outside facility prior to knowledge of the patient’s additional tumor burden. Once the liver biopsy report was available and the case was reviewed, the differential diagnosis changed significantly to include not only a primary breast adenocarcinoma (in which case the patient would have had synchronous primaries) but also primary NENB with metastases elsewhere, as well as metastatic NET to the breast from the gastrointestinal tract or somewhere else in the body. The distinction between these was made based upon the immunohistochemical workup of the breast mass.

Initial examination of the breast biopsy showed a morphologic pattern that can be seen in primary breast adenocarcinomas but is also reminiscent of neuroendocrine differentiation. Tumor cells were strongly and diffusely immunoreactive for synaptophysin and chromogranin, confirming their neuroendocrine nature. Both breast and liver tumors were immunoreactive for GATA3, and the breast tumor showed strong staining for ER. Metastases to the breast from gastrointestinal primary NETs are not uncommon and are usually to the ductal tissue [4]. GATA3, ER positivity together with ipsilateral axillary lymphadenopathy support a primary breast tumor.

The differential of tumors of the breast with neuroendocrine differentiation has been recently refined by the World Health Organization (WHO) to include a NET, neuroendocrine carcinoma (NEC), invasive carcinoma of no special type with neuroendocrine differentiation, as well as certain distinct tumor types such as solid papillary carcinoma and hypercellular mucinous carcinoma [5]. The latter two were ruled out by the lack of distinct morphology seen in those entities. NEC is an invasive carcinoma with high-grade morphology of either small cell or large cell type, which was not noted in the specimen. Breast NET is an invasive tumor with low to intermediate nuclear grade and neuroendocrine morphology supported by immunohistochemical expression of chromogranin and/or synaptophysin. The distinction between an invasive carcinoma of no special type with neuroendocrine features and a neuroendocrine neoplasm is based upon the proportion of neuroendocrine pattern present: only those tumors with greater than 90% of a neuroendocrine pattern should be considered neuroendocrine neoplasm [5].

In our case, the neuroendocrine morphology and immunohistochemical staining were seen in the entire tumor sampled. NETs of the visceral organs most commonly originate from broncho-pulmonary, gastrointestinal, or pancreaticobiliary tracts and are frequently metastatic at presentation. Metastases of NENB to the viscera are uncommon but well documented [1]. Determination of site of origin is aided by immunohistochemistry. GATA3 has a high sensitivity for luminal breast cancers, including NENB [6]. On the other hand, it is usually negative in well-differentiated NET of the lung, pancreas, and small intestine [7].

NENB are rare and historically have been poorly defined, thus complicating their characterization, diagnosis, and treatment. Neuroendocrine differentiation in breast tumors was first described in 1963 by Feyrter and Hartmann [8], and since then there have been multiple classification systems leading to variable incidences and complicated systematic trials of therapy. In the most recent WHO classification and following a consensus conference between the WHO and the International Agency for Research on Cancer that aimed to create a uniform classification framework for neuroendocrine neoplasms at any anatomic site, NENBs are now classified as either NET - well-differentiated and poorly differentiated, or NEC [5,9]. NETs in the breast are invasive tumors with low to intermediate nuclear grade, variable architecture, granular (‘’salt and peppery”) chromatin, and diffuse immunoreactivity for neuroendocrine markers. About 50% of them express chromogranin A and 16% express synaptophysin [10]. In NET of the breast, unlike NETs elsewhere in the body, grading is assessed per the same Nottingham grading system used in other breast carcinomas. Mitotic count is part of this grading. While ki-67 can be assessed as part of the molecular classification scheme and a marker of aggressive behavior, it is not a part of the formal grading in NENB. Breast NECs are invasive tumors with high-grade neuroendocrine morphology; these may be composed of either small cells or large cells, and more than two-third are immunoreactive for chromogranin and synaptophysin. Other invasive carcinomas of the breast can show neuroendocrine differentiation; the distinction between them and NETs is that NETs have greater than 90% neuroendocrine morphology and immunohistochemical staining. Finally, neuroendocrine differentiation is frequently seen in certain morphologically distinct tumors, particularly solid papillary carcinoma and the hypercellular subtype of mucinous carcinoma. These tumor types retain their own identity and are not classified as NENB [5].

There are several hypotheses but no consensus on the histogenesis of NENB. One proposal is of early divergent differentiation in breast cancer stem cells into both neuroendocrine and epithelial lineage, which explains the mixed nature of many NENB to include both endocrine and exocrine cells [11]. Moreover, this is consistent with molecular studies showing a clonal relationship of neuroendocrine cells in NENB with the intraductal epithelial tumor in breast tissue [12]. Gene expression profiling has demonstrated that NENB belongs to either the luminal subtype or the basal subtype [13,14]. Due in part to the varying definitions for neuroendocrine neoplasms of the breast over time, the estimated incidence ranges from <1% to 20% [11,15]. Because the prognostic significance of neuroendocrine differentiation in breast carcinoma is still debated [2,10] and there is no clinical relevance of neuroendocrine differentiation in and of itself, routine staining of breast tumors with neuroendocrine markers is not recommended [5]. Given the occasional subtlety of the neuroendocrine morphology and a lack of immunostaining in many potential cases, recognition of NENB may be difficult, and the true incidence of these tumors is unknown. In cases that are confined to the breast, there is no clinical significance to identify the neuroendocrine differentiation; however, in cases such as this patient where there is tumor burden elsewhere determination of the histogenesis and relationship of each site to identify the primary tumor site of origin can be critical.

Most of the scientific evidence for NENB treatment is based on clinical trials performed for metastatic primary gastroenteropancreatic (GEP)-NETs. Although several treatment regimens have been associated with antitumor activity in NET, there have been no consensus guidelines on the optimal chemotherapy regimen for advanced NET. In the recent past, peptide receptor radiotherapy with radiolabeled somatostatin analogs has been studied in a phase-3 trial in patients with advanced somatostatin receptor-positive, well-differentiated metastatic midgut NETs. Treatment with 177Lu-Dotatate resulted in markedly improved 20-month progression-free survival (65% vs 18% in the control group) and a significantly higher response rate (18%; p<0.001) compared to somatostatin analogs (3%) [16]. Investigators are studying radioligands targeting peptide receptor treatments including gastrin-releasing peptide receptor/bombesin receptor 2 (GRPR), somatostatin receptor 2 (SSTR2), and Chemokine C-X-C Motif Receptor 4 (CXCR4) in breast cancer [17]. Further clinical studies are required to use these agents for imaging and treatment of NETs.

Universal management guidelines for metastatic NENB are lacking and are extrapolated from clinical trials performed in patients with GEP-NETs. CAPTEM is a novel chemotherapy combination regimen with proven efficacy in well-differentiated NETs. This combination has shown synergistic properties when tested on two NET cell lines in vitro [6]. The precise mechanism of synergism is unclear, though is thought to be mediated by apoptosis within the neuroendocrine lineage. CAPTEM was first studied in patients with metastatic pancreatic NETs and achieved an objective radiographic response rate of 70%, median PFS of 18 months, and two-year overall survival of 92%, which was confirmed in later studies [18]. CAPTEM was associated with high response rates (29.1%) in patients with advanced NETs [19]. Kunz et al. conducted a randomized phase-II trial comparing temozolomide with CAPTEM in patients with advanced pancreatic NETs. Results showed significant improvement in median PFS (22.7 months for CAPTEM vs. 14.4 months for temozolomide; HR = 0.58, p = 0.023) and median OS (38 months for temozolomide vs not reached for CAPTEM; HR = 0.41, p = 0.012) [20]. This regimen is well-tolerated with rare grade 3 and 4 toxicities [18]. Notably, palmar-plantar dysesthesia was reported in 5.5% of patients, which our patient also experienced.

## Conclusions

Primary NENB is an uncommon entity. Neuroendocrine differentiation in breast tumors can be seen in primary breast NETs, NECs, invasive carcinomas of no special type with neuroendocrine differentiation, solid papillary carcinoma, certain mucinous carcinomas, and metastases of NETs to the breast. NENBs are unusual but should be considered in the differential diagnosis of breast tumors. While the recognition of neuroendocrine differentiation may not be clinically significant in tumors confined to the breast, in patients with NETs in multiple organs, confirmation of histogenesis of each is important to identify the appropriate primary site of origin. Strong clinico-radiologic correlation and multidisciplinary communication are vital to making this distinction. Somatostatin-receptor imaging and careful clinical and histopathologic evaluation are crucial to NENB diagnosis and differentiating it from extramammary origin. CAPTEM shows promise in the treatment of metastatic NENB and other metastatic NETs. Our patient showed a good response to systemic treatment and continues to enjoy a good quality of life.
